# Clinical outcomes in central nervous system solitary-fibrous tumor/hemangiopericytoma: a STROBE-compliant single-center analysis

**DOI:** 10.1186/s12957-022-02619-w

**Published:** 2022-05-10

**Authors:** Yang Yu, Yu Hu, Liang Lv, Cheng Chen, Senlin Yin, Shu Jiang, Peizhi Zhou

**Affiliations:** Department of Neurosurgery, Pituitary Adenoma Multidisciplinary Center, West China Hospital of Sichuan University, No. 37, Guoxue Alley, Chengdu, 610041 China

**Keywords:** Solitary fibrous tumor, Hemangiopericytoma, Quality of life, Prognosis, Central nervous system

## Abstract

**Background:**

Solitary fibrous tumor (SFT) and hemangiopericytoma (HPC) are rare mesenchymal tumors in the central nervous system with a high tendency to relapse, having a significant impact on quality of life (QoL). Due to the rarity of intracranial SFT/HPC, the prognostic factors and optimal treatment remain to be elucidated. Meanwhile, quality of life in patients with intracranial SFT/HPC is seldomly concerned. Thus, we aim to survey about the quality of life and underline some aspects demanding concern in intracranial SFT/HPC treatment through summarizing our case series in recent ten years.

**Methods:**

Patients with intracranial SFT/HPC who underwent surgical resection from January 2009 to June 2019 were included in the study. Clinical features, such as age, gender, and resection extent, were collected. The EuroQol Five Dimensions Questionnaire (EQ-5D) was used to assess the patients’ quality of life (QoL). Prognosis factors related to progression-free survival (PFS) and overall survival (OS) were also evaluated.

**Results:**

Thirty-six patients with a mean follow-up period of 61.6 months (range 13–123 months) were included in this study. Sixteen (44.4%) patients achieved gross total resection (GTR). Fourteen patients (38.9%) with tumor progression experienced adjuvant radiotherapy (11.1%) or Gamma Knife surgery (GKS, 27.8%). According to the 2016 WHO classification, there were 6 (16.7%) grade I SFT/HPC, 11 (30.5%) grade II SFT/HPC, and 19 (52.8%) grade III SFT/HPC. The PFS and OS were 29 months (range 4–96 months) and 38 months (range 4–125 months). The median EQ5D-3 L tariff with or without progression was 0.617 (95% CI 0.470–0.756) and 0.939 (95% CI 0.772–0.977) respectively. Gross total resection (GTR, *p* = 0.024) and grade I SFT/HPC (*p* = 0.017) were significantly associated with longer PFS. In multivariate analysis, GTR (HR 0.378, 95% CI 0.154–0.927) and adjuvant therapy (HR 0.336, 95% CI 0.118–0.956) result in significantly longer PFS in patients with SFT/HPC.

**Conclusions:**

Patients underwent GTR and adjuvant therapy had longer PFS. Similarly, patients with lower WHO grade had relatively longer PFS. Therefore, GTR is advocated for the treatment of SFT/HPC. And adjuvant therapy such as GKS could be an alternative treatment for patients who underwent STR or with tumor progression. Further, the QoL decreased in patients with tumor progression and metastasis, and more attention is demanded to the QoL of intracranial SFT/HPC patients.

## Introduction

Solitary fibrous tumors (SFTs) were first reported as local mesenchymal neoplasms in the visceral pleura, but extrathoracic sites are increasingly being recognized then after. Intracranial SFTs are rare and were first described by Carneiro et al. in 1996 [1]. They have been generally considered benign tumors that originated predominantly from thick collagen bands [2]. Hemangiopericytomas (HPCs) are also rare mesenchymal tumors that exhibit similar clinical, radiological, and histological features as SFTs [3]. However, SFTs and HPCs show distinct biological behaviors: SFTs are generally slow-growing indolent tumors with rare recurrence and metastasis after total resection, while HPC grows aggressively with frequent recurrence and metastasis following total resection [3, 4]. Due to their distinct biological behaviors, the combination of intracranial SFTs and HPCs into a single entity was delayed. However, recent pathological findings of their shared genetic etiology prompted the World Health Organization (WHO) to classify the two types of tumors into one combined entity in 2016 [5–8].

Several previous studies explored the prognostic factors of intracranial SFT/HPC, which mostly underlined the extent of resection (EOR) as a major factor for tumor recurrence and metastasis [9–16]. Other factors, including WHO classification [9, 15], adjuvant therapy [16–20], age [21, 22], location, and size of tumor [23] had also been reported that related to patient’s progression-free survival (PFS) and overall survival (OS). However, due to the rarity of intracranial SFT/HPC, little is known about the optimal treatment and prognostic factors, and our current knowledge about SFT/HPC is mainly derived from small case series [4, 9, 11, 14]. Moreover, although it is well-known that the quality of life (QoL) of patients who suffered other intracranial tumors dramatically decreased, especially when tumor recurrence and metastasis occurred, little attention has been paid to the QoL of intracranial SFT/HPC patients [24–26].

Thus, we retrospectively reviewed our department’s intracranial SFT/HPC cases from January 2009 to June 2019 to investigate the clinical characteristics, QoL, and prognosis factors related to PFS and OS.

### Patients and methods

From January 2009 to June 2019, patients who underwent surgical resection for intracranial SFT/HPC in West China Hospital, Sichuan University, were identified retrospectively. Two experienced pathologists confirmed the diagnosis of SFT/HPC according to the 2016 WHO classification of tumors of the central nervous system (CNS). Patients without complete information and who refused to be followed were excluded. This study, registered in ChiCTR (ChiCTR2000029718), was approved by the Ethics Committee of West China Hospital and informed consent was obtained from the patient. It adheres to the tenets of the Declaration of Helsinki.

Thirty-nine patients were initially identified for postoperative pathology results of head SFT/HPC. Two patients were excluded for incomplete information and another for the extracranial lesion. Electronic medical record (EMR) was used to collect specific patient information by three individual investigators with final cross-check, including demographic characteristics, location, the largest diameter of tumor (LD), EOR, pathological findings, adjuvant therapies, recurrence and metastasis, and follow-up conditions. The EOR was separately confirmed by two investigators referring to operation records and postoperative magnetic resonance imaging (MRI) findings. Gross total resection (GTR) or subtotal resection (STR) was defined as previously described [2]. Specifically, macroscopically complete tumor resection with or without removal or coagulation of affected dura and underlying bone was classified as GTR. Whereas subtotal tumor resection, decompression, and biopsy were defined as subtotal resection. Progression was defined as recurrence with the lesion reappeared after GTR or the enlargement of residual tumor and metastasis with the extracranial presence of SFT/HPC lesion.

**Fig. 1:**
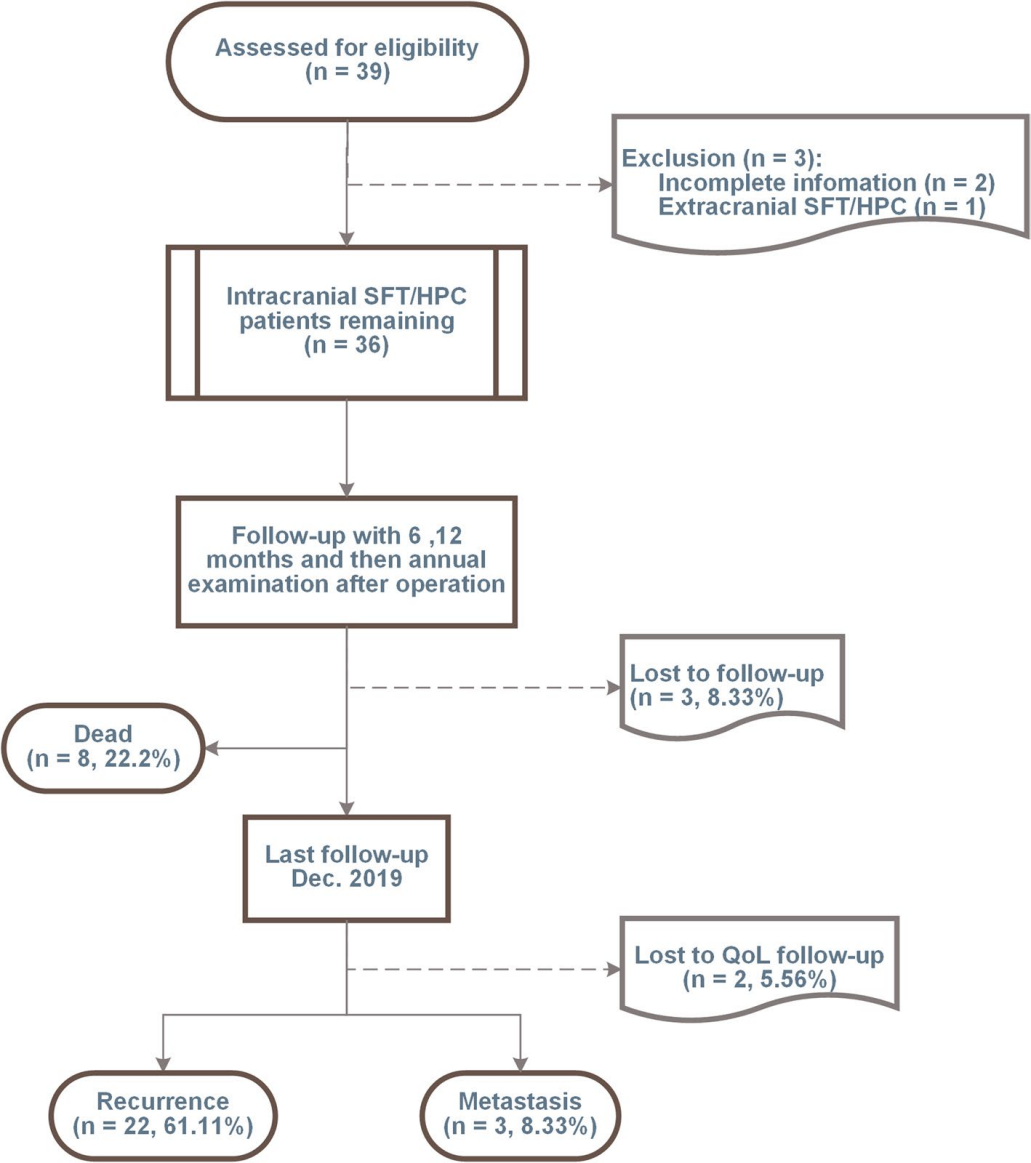
The flowchart of enrollment and follow-up. Initially, 39 patients were identified according to postoperative pathology results of SFT/HPC. Then, two patients were excluded for incomplete information and one for the extracranial lesion. Three patients lost response before the last follow-up for QoL. Moreover, eight patients had been dead before the last follow-up. Two patients did not respond to the investigation of QoL at the last follow-up

All patients were followed up after hospitalization and evaluated 6 and 12 months after surgery. Then annual examinations were conducted for life. The follow-up period was calculated as the duration from surgery to death or until December 2019 for surviving patients. Follow-up information was obtained from outpatient EMR and telephone interviews. At the last follow-up, the EuroQol Five Dimensions Questionnaire (EQ-5D) was used to evaluate the patient’s QoL through routine medical appointments or telephone interviews. The enrollment and follow-up process is shown in Fig. 1. The EQ-5D tariff was calculated through the time-trade-off method reported in a previous Chinese study [27].

### Statistical analysis

Data analysis was performed by IBM SPSS 25 Software (IBM, New York, USA). Fisher’s exact test and independent-sample *T*-test were separately used to explore classified and numeric variables. Moreover, the Kruskal-Wallis test was used to analyze the WHO classification among different groups. EQ-5D tariff was tested by using the Mann-Whitney *U* test. Univariate analysis was conducted with the Kaplan-Meier method for PFS and OS, using the Log-rank test for determining its significance. Multivariate Cox regression analysis was performed using significant factors in univariate analysis. It was considered statistically significant when a *p*-value was less than 0.05.

## Results

### Patient characteristics

**Fig. 2:**
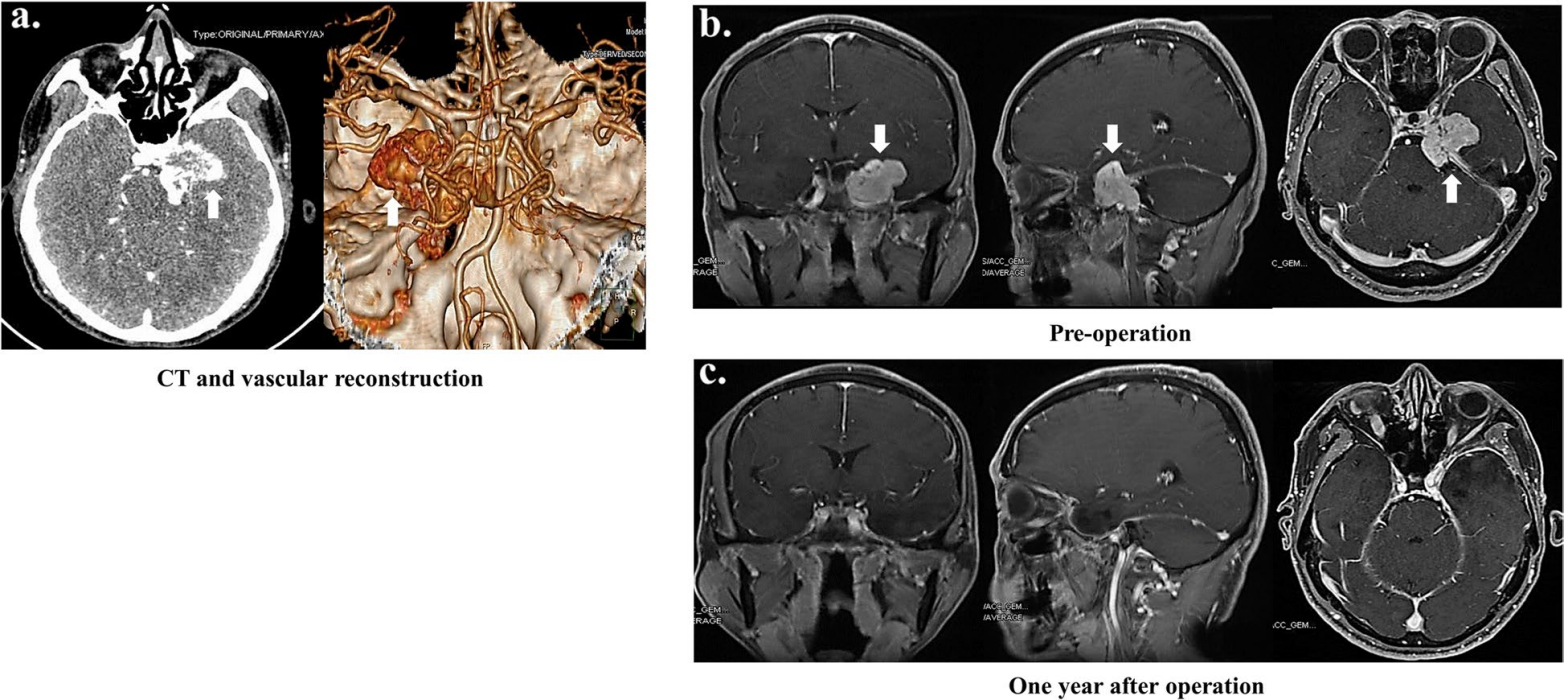
Representative CT and MRI images before and after operation. **a** Preoperative CT and vascular reconstruction images exhibited the SFT/HPC lesion in the left middle cranial fossa. This irregular high-density mass showing heterogeneous enhancement had unclear boundaries and destroyed the surrounding skull. And this lesion partially enveloped the left internal carotid artery segments C3-C4. **b** The preoperative enhanced T1WI images were displayed. The mass was located in the left middle cranial fossa, with unclear boundaries and heterogeneous enhancement. The lesion protruded locally into the left pontine corner cistern, partially compressed the left temporal lobe and brainstem and enveloped the C3-C4 segments of the left internal carotid artery. **c** The enhanced T1WI images of 1-year past operation were exhibited. The area of the original lesion showed postoperative changes without significant enhancement

Thirty-six intracranial SFT/HPC patients were included in this study from 2009 to 2019 (Table 1). There were 22 male (61.1%) and 14 female (38.9%) patients, with a median age of 47 years ranging from 13 to 75 years. Twenty-nine tumors (80.5%) were located supratentorial, and seven (19.5%) were infratentorial locations. The maximal tumor diameter ranged from 2.0 cm to 10.0 cm, with a median diameter of 5.0 cm. Sixteen (44.4%) patients underwent GTR. The PFS and OS were 29 (range, 4 to 96) months and 38 (range, 4 to 125) months. Fourteen patients (38.9%) with tumor recurrence or metastasis were further treated with adjuvant radiotherapy (11.1%) or Gamma Knife surgery (GKS, 27.8%). According to the 2016 WHO classification, there were 6 (16.7%) grade I SFT/HPC, 11 (30.5%) grade II SFT/HPC, and 19 (52.8%) grade III SFT/HPC. The mean follow-up period was 61.6 months (range 13–123 months). Among these patients, eight (22.2%) individuals were dead in 4 to 84 months (median, 42 months) after the first operation. Tumor recurrence occurred in 22 (61.1%) patients from 6 to 68 months (median, 25.5 months) after the first surgery. Three (8.33%) patients suffered from extracranial tumor metastases, including the lung (2 patients), ovary (1 patient), and pelvic bone (1 patient). In Fig. 2a, preoperative CT and vascular reconstruction images of typical SFT/HPC patients were shown. Also, preoperative (Fig. 2b) and postoperative (Fig. 2c) enhanced T1WI MRI images were displayed.

**Table 1:** Characteristic of 36 patients diagnosed with STF/HPC

Aspects	Totality
Gender (M/F)	22/14
Age	47 (13, 75)
Tumor location (no. [%])	
Supratentorial	29 (80.5)
Infratentorial	7 (19.5)
Tumor size in cm (no. [%])	
< 5.0	17 (47.2)
≥ 5.0	19 (52.8)
EOR (no. [%])	
GTR	16 (44.4)
STR	20 (55.6)
Adjuvant therapy (no. [%])	
Radiotherapy	4 (11.1)
GKS	10 (27.8)
WHO classification (no. [%])	
Grade I	6 (16.7)
Grade II	11 (30.5)
Grade III	19 (52.8)

### Quality of life

At the last follow-up, twenty-three patients were tested by EQ5D-3 L. The median EQ5D-3 L tariff of the patients with or without progression were 0.617 (95% CI: 0.470–0.756) and 0.939 (95% CI: 0.772–0.977), respectively. The EQ5D-3 L tariff significantly decreased in patients whose disease progressed (*p* = 0.002). Further, the dimension for pain or discomfort in patients with tumor recurrence or metastasis (median, 0.092; range, 0–0.236) was significantly higher than non-progression patients (median, 0; range, 0–0.092), with a *p*-value of 0.049. Moreover, patients with progression obtained higher score (*p* = 0.026) in dimension of negative emotion (median 0.043, range 0–0.205), compared with non-progression patients (median, 0; range, 0–0.086). Consequently, QoL in intracranial SFT/HPC patients significantly declined when the tumor relapsed. Intracranial patients with tumor recurrence and metastasis were intended to suffer body pain and stay in negative emotion frequently.

### Univariate analysis of the factors related to tumor PFS and OS

**Fig. 3:**
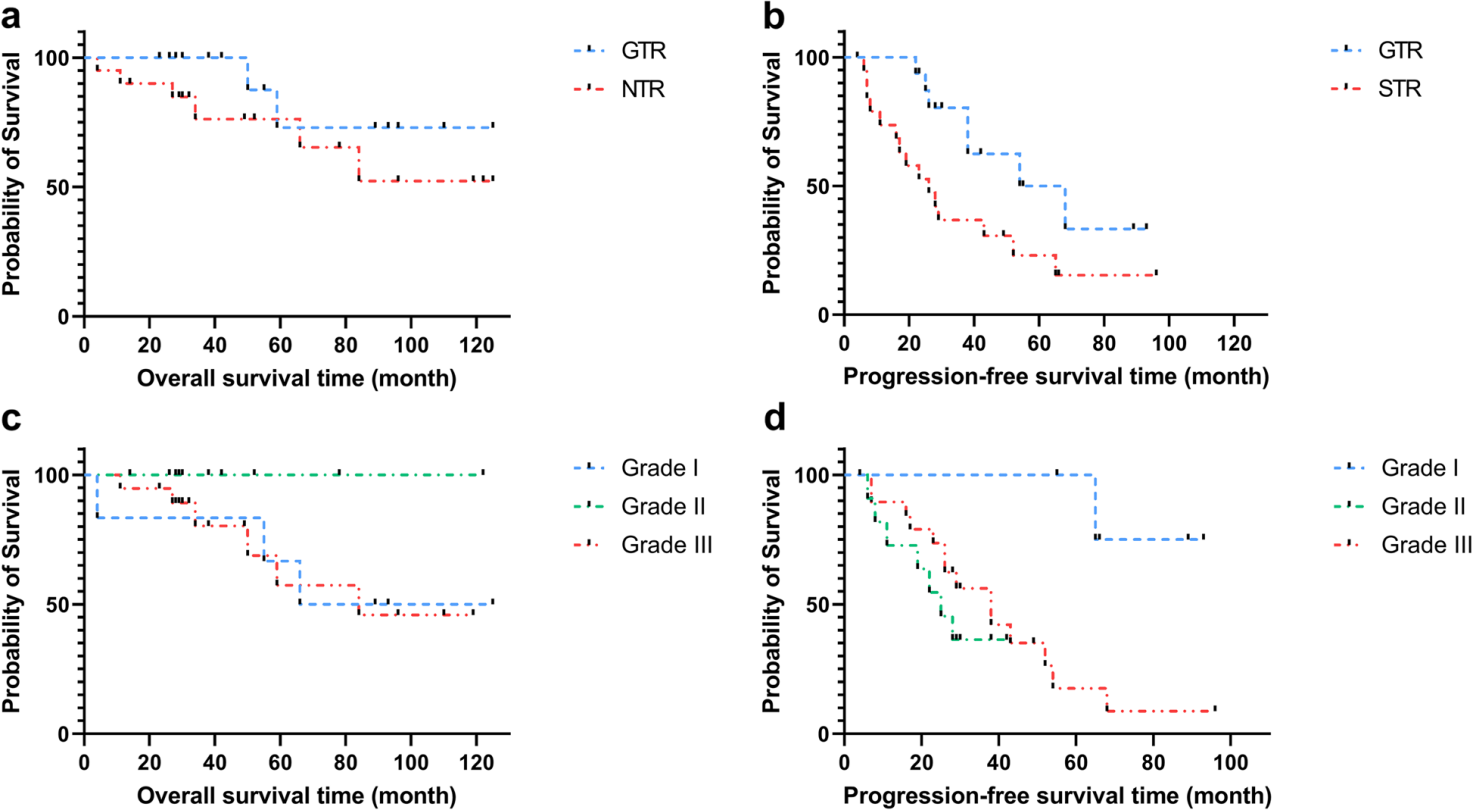
Kaplan-Meier survival curves display over all-survival and progression-free survival with different EOR and WHO grade. Figures exhibited EOR (**a**, **b**) and WHO grade (**c**, **d**)-related overall-survival and progression-free survival. EOR and WHO grade in PFS were statistically significant with *p*-value equaling 0.024 and 0.017. GTR, gross total resection; STR, subtotal resection

Several prognosis factors were detected for disease progression, involving LD (< 5.0 cm and ≥ 5.0 cm), EOR, adjuvant therapy, and WHO grade. Consequently, EOR and WHO grade displayed statistical significance (Table 2). Patients who underwent GTR obtained longer PFS (60.7 vs. 36.7 months, *p* = 0.024) than those who underwent STR patients. There is no significant difference in OS between the GTR and STR groups (92.7 vs. 88.6 months, *p* = 0.270) (Fig. 3). Moreover, patients with Grade I SFT/HPC had longer PFS (86.0 months) than those with Grade II (26.1 months) and Grade III (40.5 months) (*p* = 0.017).

**Table 2:** Univariate analysis for EOR, adjuvant therapy, and WHO classification

	Overall survival		Progression-free survival	
	Median	95% CI	Median	95% CI
LD (cm)		0.553		0.164
< 5.0	91.52	(65.30, 117.73)	61.31	(40.24, 82.37)
≥ 5.0	70.23	(53.28, 87.18)	42.04	(27.29,56.80)
EOR^a^		0.270		0.024
GTR	92.68	(72.65, 112.72)	60.66	(44.89, 76.44)
STR	88.57	(65.59, 111.55)	36.73	(22.60, 50.85)
Adjuvant therapy		0.057		0.06
With AT	99.98	(83.45, 116.53)	51.46	(39.09, 63.84)
Without AT	30.38	(21.14, 39.61)	22.14	(10.33, 33.95)
WHO grade^a^		0.254		0.017
Grade I	92.54	(54.61, 130.48)	86.00	(74.12, 97.88)
Grade II	–	–	26.09	(18.04, 34.14)
Grade III	81.81	(69.58, 103.04)	40.46	(28.46, 52.46)

^a^EOR and WTO grade were statistically significant in PFS

### Multivariate analysis of the factors related to PFS

According to the outcomes of univariate analysis, EOR, adjuvant therapy, and WHO classification were used for multivariate analysis because of the limited sample sizes of this study. As shown in Table 3, EOR (HR: 0.378; 95% CI: 0.154, 0.927) and adjuvant therapy (HR: 0.336; 95% CI: 0.118, 0.956) were statistically significant for patients’ PFS with SFT/HPC.

**Table 3:** Multivariate analysis for EOR, adjuvant therapy, and WHO classification of PFS

Aspects	*p*-value	HR	95% CI of OR
Adjuvant therapy^a^	0.041	0.336	(0.118, 0.956)
EOR^a^	0.034	0.378	(0.154, 0.927)
WHO grade	0.096	1.663	(0.914, 3.025)

^a^EOR and adjuvant therapy were statistically significant in multivariate analysis

## Discussion

This study first investigated the QoL of patients with intracranial SFT/HPC and found that the EQ5D-3 L index value decreased significantly in patients with progression. Moreover, we further explored several prognosis factors related to the patient’s PFS and OS. The multivariate analyses suggested EOR and adjuvant therapy influenced patient PFS and OS in intracranial SFT/HPC.

Quality of life plays an important role in long-term surviving patients, but little attention has been paid to patients with intracranial SFT/HPC. EQ-5D is a valid and convenient implementation to survey QoL of patients who suffered from the tumor with satisfactory reliability and validity [28, 29]. Unsurprisingly, the overall QoL of patients with progression deteriorated significantly, suggesting more attention was demanded in managing intracranial SFT/HPC patients. Specifically, patients with recurrence or metastasis were burdened with much more pain and pressure, in contrast with seldom pain management and psychological support under present treatment.

In accord with previous studies, our results also demonstrated that patients with STR are more likely to suffer from tumor recurrence or metastasis. Concretely, Zeng et al. had reported that the recurrence rate and metastasis rate were respectively 59.09% (13/22) and 50.0% (3/6) in none GTR patients with a GTR rate of 74.13% (43/58) [3]. Furthermore, in other case series, the GTR rates varied from 46.7 to 86.9%, and the rate of recurrence or metastasis was 77.8 to 85.71% in none GTR patients [9, 11, 30, 31]. For the long-term follow-up, patients who obtained GTR were observed with longer PFS and OS [3, 11]. Our results analogously exhibited that the patients who performed GTR got longer PFS.

Concerning WHO classification in CNS, most researchers considered the classification as a crucial prognostic factor [2, 9, 15]. Tumor with higher-grade and nuclear atypia was more likely to relapse, but some studies demonstrated that “benign-looking” WHO grade I SFT/HPC could recur malignant degeneration [32]. Other pathologic classifications could be utilized to identify prognosis factors for intracranial SFT/HPC patients [15]. Our results showed that the WHO classification was significant for PFS in univariate analysis, while it was not statistically significant for OS and both in multivariate analysis, similar to the previous publication [9]. The limited sample size probably caused counterintuitive results.

Patients with other postoperative treatments, such as external beam radiotherapy and Gamma Knife, probably had a better prognosis [3, 11, 14, 19, 20]. Other studies showed radiotherapy could significant promote OS (HR: 0.02; 95% CI: 0.00–0.31) and cause-specific survival (HR: 0.02; 95% CI: 0.00–0.45) in multivariate analysis [19]. Our results exhibited similar results that adjuvant therapy could extend PFS. Therefore, adjuvant therapy enabled extended PFS and OS, which could be used as remedial measures when GTR could not perform and metastasis occurred.

Despite the retrospective nature, we used several methods to avoid recalling bias, including catching information from medical records by different researchers. Moreover, we can see that SFT/HPC is a rare disease, so most current studies contain 10 to 64 patients, encountering the problem of a small sample size like us. Some studies reported relatively larger samples but ran a comparatively long period, which made it difficult to maintain the comparability in the cohort, such as the bias due to the update of treatment. Also, as a single-center study, we had to recognize that selection bias was inevitable. However, patients in this study were from several provinces in China, which relatively ameliorated this influence. In the further study, we could use some quantitative synthesis methods or set up multicenter cooperation to figure out this problem.

## Conclusions

Intracranial SFT/HPC are rare mesenchymal tumors with a high relapse tendency, shortening the OS and decreasing the QoL of patients, particularly those with tumor progression and metastasis. Several factors are relevant to the prognosis, such as EOR, adjuvant therapy, and WHO grade. GTR without causing new neurological deficits is advocated for the treatment of SFT/HPC. And adjuvant therapy such as GKS could be an alternative treatment for patients who underwent STR or with tumor progression.

## Data Availability

The datasets of the current study are available from the corresponding author on reasonable request.

## Abbreviations

CNS: Central nervous system
EQ-5D: EuroQol Five Dimensions Questionnaire
EMR: Electronic medical record
EOR: Extent of resection
GKS: Gamma knife surgery
GTR: Gross total resection
HPC: Hemangiopericytoma
LD: Largest diameter
MRI: Magnetic resonance imaging
OS: Overall survival
PFS: Progression-free survival
QoL: Quality of life
SFT: Solitary fibrous tumor
STR: Subtotal resection
WHO: World Health Organization
